# Soil-transmitted helminth infections and physical fitness in school-aged Bulang children in southwest China: results from a cross-sectional survey

**DOI:** 10.1186/1756-3305-5-50

**Published:** 2012-03-16

**Authors:** Peiling Yap, Zun-Wei Du, Ran Chen, Li-Ping Zhang, Fang-Wei Wu, Jian Wang, Xue-Zhong Wang, Hui Zhou, Xiao-Nong Zhou, Jürg Utzinger, Peter Steinmann

**Affiliations:** 1 Department of Epidemiology and Public Health, Swiss Tropical and Public Health Institute, P.O. Box, CH-4002 Basel, Switzerland; 2 University of Basel, P.O. Box CH-4003 Basel, Switzerland; 3 Helminthiasis Division, Yunnan Institute of Parasitic Diseases, Pu'er 665000, People's Republic of China; 4 Menghai Center for Diseases Control and Prevention, Menghai 666200, People's Republic of China; 5 Sichuan Institute of Parasitic Diseases, Chengdu 610041, People's Republic of China; 6 National Institute of Parasitic Diseases, Chinese Center for Diseases Control and Prevention, Shanghai 200025, People's Republic of China

## Abstract

**Background:**

Chronic soil-transmitted helminth (STH) infections have been associated with reduced physical fitness, but available evidence is limited. The aim of this cross-sectional survey was to assess the feasibility of measuring children's physical fitness and to relate it to STH infections. Our study was carried out among school-aged children of the Bulang ethnic group in rural southwest People's Republic of China (P.R. China). Standardized, quality-controlled methods were employed to determine STH infections (Kato-Katz technique), haemoglobin levels, anthropometry (body weight and height) and physical fitness (20-m shuttle run test).

**Results:**

A compliance of 87% suggested good acceptance of the methods used. Among 69 children with complete data records, infection prevalence of *Trichuris trichiura, Ascaris lumbricoides *and hookworm were 81%, 44% and 6%, respectively. The maximum volume of oxygen that can be utilized within 1 min during exhaustive exercise (VO_2 _max estimate) of *T. trichiura*-infected children was 1.94 ml kg^-1 ^min^-1 ^lower than that of their non-infected counterparts (*P *= 0.005). Until exhaustion, *T. trichiura*-infected children had completed 6.14 20-m laps less (*P *= 0.004). Additionally, the mean VO_2 _max estimate of stunted children was lowered by 1.63 ml kg^-1 ^min^-1 ^(*P *= 0.002) and they completed 5.32 20-m laps less (*P *= 0.001) compared to children of normal stature. No significant association between stunting and infection with any STH species could be established.

**Conclusions:**

Implementation of physical fitness tests in rural, resource-constraint settings is feasible. The physical fitness of children who are stunted or infected with STHs, particularly *T. trichiura*, is significantly impaired. We have launched a larger study and will determine the dynamics of school-aged children's physical fitness over a 7-month period after administration of anthelminthic drugs.

## Background

More than 1 billion people are parasitized by soil-transmitted helminths (STHs), namely *Ascaris lumbricoides, Trichuris trichiura *and the hookworms (*Ancylostoma duodenale *and *Necator americanus*) [1-4]. Taken together, STHs represent the most prevalent parasitic infection of mankind, but determining their exact geographical distribution, morbidity and global burden has proved difficult [5,6]. The highest infection rates are observed in the developing world. Control programmes in these regions emphasize preventive chemotherapy, i.e. repeated administration of anthelminthic drugs to entire at-risk populations, particularly school-aged children [3,7,8]. Recognized common symptoms include abdominal pain, diarrhoea, anaemia, growth retardation and cognitive impairment. Hosts often carry more than one species simultaneously and may suffer from additive and/or multiplicative morbidity outcomes [9,10].

In the global burden of disease (GBD) study carried out between 2000 and 2004, high-intensity infections with STHs were given a zero disability weight (DW), while the cognitive impairment which can result from such infections was assigned DWs ranging from 0.024 to 0.463 [on a scale from 0 (perfect health) to 1 (death)], depending on the helminth species [11,12]. Other recognized disabilities included were massive dysentery syndrome for *T. trichiura *(DW 0.116), anaemia for hookworm and intestinal obstruction due to *A. lumbricoides *infections (each with a DW of 0.024). From these estimates it becomes evident that according to the current consensus, individuals infected with STHs usually do not experience overt morbidity or life-threatening manifestations. However, the chronic under-nourishment [13,14] and impaired physical and mental development [15] associated with such infections in childhood arguably prevent populations in which STH infections are pervasive to realize their full potential. Together with other unfavourable conditions, this threatens to perpetuate their entrapment in the vicious cycle of poverty and poor health [16].

Two decades ago, Stephenson and colleagues reported that the physical fitness, as determined by the Harvard step test (HST) [17], of Kenyan school boys infected with STHs had improved 7 weeks after the administration of a single dose of albendazole [18]. In another study by this group, which looked at physical fitness 4 months post-treatment, similar results were obtained [19]. Subsequently, little scientific inquiry was made, but recently, the question was re-visited in a cross-sectional survey carried out in Kenya. The study confirmed that hookworm infection is negatively correlated with fitness scores in girls and teenage women aged 5-18 years. The reduced physical fitness was attributed to anaemia and stunting resulting from chronic hookworm infection [20]. However, a cross-sectional survey carried out in Côte d'Ivoire, with the aim of determining the effect of STHs and schistosome infections on physical fitness, failed to demonstrate a correlation in children aged 7-15 years [21]. Both studies employed the 20 m shuttle run test to measure fitness [22].

The objective of the present study was to contribute to the small evidence-base regarding the effect of helminth infections on physical fitness of school-aged children. The study was designed as a cross-sectional survey, assessing the technical and operational feasibility of measuring physical fitness among school-aged children in rural southwest P.R. China where STH infections are widespread.

## Methods

### Study sites

The study was carried out in the primary schools of three villages inhabited by members of the Bulang ethnic minority group, located in Menghai county, Xishuangbanna Dai autonomous prefecture, Yunnan province, P.R. China, from May to June 2011. The Bulang speak their own traditional language but the younger generation is increasingly learning to speak and write Mandarin Chinese. However, current literacy rates are still low, with most of the adults being illiterate or only having obtained primary education. The Bulang traditionally build their villages in mountainous regions, while the fertile plains are dominated by another minority, namely the Dai.

The area enjoys a subtropical climate characterized by heavy rainfall during the summer monsoon season and mild winter months. The Bulang rely on agriculture for income generation, with Pu'er tea as the most important cash crop. Livestock breeding is limited and pigs are the main domestic animals. General living standards are low. Systematic STH control activities have been implemented in the study villages since 2009 (periodic distribution of albendazole to the entire population, construction of family latrines in Manguo new village).

The three study villages are (i) Manguo new village (geographical coordinates: 21°45'09.01″ N latitude and 100°18'47.20″ E longitude); (ii) upper Nanwen (21°46'02.15″ N and 100°23'50.61″ E); and (iii) lower Nanwen (21°46'34.02″ N and 100°23'56.89″ E), all located at high altitudes (1550-1650 m above sea level). These villages are comparable among themselves and to other Bulang villages in terms of socio-demography, general level of development, infrastructure and other features. Important characteristics of Bulang villages have been described in detail in previous publications pertaining to the epidemiology and control of STHs in Bulang communities [23-26].

### Study design

The present study pursued a cross-sectional design. Participants submitted stool samples for subsequent parasitological examination in a laboratory using light microscopy. Anthropometric indicators (height and weight), haemoglobin and family-level socio-economic conditions were assessed. Children's physical fitness was determined based on their performance in a 20-m shuttle run test.

### Ethical considerations

The study protocol was approved by the institutional research commission of the Swiss Tropical and Public Health Institute (Basel, Switzerland). Ethical clearance was granted by the ethics committee in Basel (EKBB, reference no. 144/11) and the Academic Board of the National Institute of Parasitic Diseases, Chinese Center for Disease Control and Prevention (Shanghai, P.R. China).

The village chiefs, doctors, teachers and study participants were briefed on the study aims and procedures. Children who were absent from school or suffering from any major illness were excluded from the study. All other pupils aged 8-15 years were invited to participate. Since most parents/guardians are illiterate, oral informed consent was sought. Children could withdraw from the study anytime without further obligations. Upon registration, each study participant was given a unique ID number. In all subsequent steps, this number was used to ensure that all results were kept anonymous. At the end of the study, sufficient albendazole to treat the entire communities was handed over to the village health authorities, who were in charge of distributing them to villagers aged 2 years and above (400 mg, single oral dose).

### Study procedures

During registration, the age of the participant and the main source of income of his/her family were recorded. Participants were given a pre-labelled (name, ID) stool collection container and encouraged to submit one stool sample the following morning. Filled containers were collected between 07:00 and 09:00 hours and transported to the County Center for Disease Control and Prevention (CDC) laboratory in Menghai city for diagnostic work-up on the same day. Each stool sample was visually inspected for the presence of *Taenia *spp. proglottids. Subsequently, duplicate Kato-Katz thick smears were prepared from each sample, and read within 60 min after preparation, under 400× magnification [27]. Stratified by helminth species, the mean number of eggs from the duplicate thick smears was multiplied by a factor 24 to obtain an estimate of the number of eggs per gram (EPG) of stool [28]. For quality control, the two slides were independently read by different technicians and the results compared. Slides were re-read if inconsistencies were detected.

Field workers blinded to the infection status of the study participants performed the anthropometric measurements. Body weight was measured (to the nearest 0.1 kg) twice using a digital scale (Model RCS-150; Nantong Xineng Ltd., Jiangsu, P.R. China), and averaged. For each measurement, the participant was asked to stand in the centre of the scale platform without shoes and sweaters. The height of each participant was also measured (to the nearest 0.1 cm) twice, and averaged. A stadiometer (Nantong Xineng Ltd, Jiangsu, P.R. China) was used and the participant stood, without shoes, with his/her back erect but shoulders relaxed against the stadiometer, while the headpiece was lowered to the crown of the head with sufficient pressure to compress the hair.

The body weight and height values were used to calculate the body mass index (BMI: defined as weight/height^2^), BMI-for-age Z score (BAZ: an indicator for wasting) and height-for-age Z score (HAZ: an indicator for stunting) [29].

For haemoglobin measurements, a fresh set of alcohol swab, safety lancet and microcuvette was used for each participant. The ear lobe was pricked and the second drop of blood taken up by the microcuvette for reading with a HemoCue^®^ AB 301 system (HemoCue^®^AB; Ängelholm, Sweden). Anaemia was defined according to WHO age-specific cut-offs: Hb < 11.5 g/dl for ages < 12 years; Hb < 12 g/dl for ages ≥ 12 years and < 15 years; Hb < 13 g/dl for males ≥ 15 years [30].

Lastly, a 20-m shuttle run test was performed. All participants were asked by the village doctor and teachers to indicate any body discomfort they might have, and the doctor stayed throughout the test to ensure that medical attention could be given immediately if required. The test was carried out between 10:00 and 12:00 hours in adherence to standard published procedures [20,22]. Participants, in groups of five, ran back and forth on a 20 m flat course, following the pace of pre-recorded sound signals (Team Bleep Test Version 1.3.1, Bitworks Design; Cheltenham, UK). Starting with a running speed of 8.5 km h^-1^, the frequency of the signals indicating 20 m intervals increased by 0.5 km h^-1 ^every min. During the test, participants were encouraged constantly to complete as many courses as possible. When a participant failed to follow the pace for two consecutive 20 m intervals, he/she was asked to stop. The running speed from the last completed interval and the total number of laps completed were recorded. The age and speed were converted into the maximum volume of oxygen that can be utilized within 1 min during exhaustive exercise (VO_2 _max estimate, expressed in ml kg^-1 ^min^-1^) with the equation put forth by Léger *et al. *[22]. Physical fitness of each participant was expressed as the VO_2 _max estimate and the number of laps completed.

### Statistical analysis

Data were entered into Excel version 2008 (Microsoft Corp.; Redmond, WA, USA), double-checked and merged into a single database for statistical analysis with STATA version 10.0 (STATA Corp.; College Station, TX, USA). The children's parasitological status was assessed in terms of prevalence, infection intensity (mean EPG) and multiparasitism (concurrent infections with more than one helminth species). Anthropometric indicators, haemoglobin concentrations, VO_2 _max estimate and the number of laps completed in the 20-m shuttle run test were expressed as means. Comparisons were made between participants infected with a particular STH species and participants not infected with that specific species. Test statistics included chi-square (χ^2^), Fisher's exact, Wilcoxon rank-sum, Kruskal-Wallis, two sample *t*-tests and multiple linear regression models, as appropriate.

## Results

### Compliance and demography of study participants

**Figure 1:**
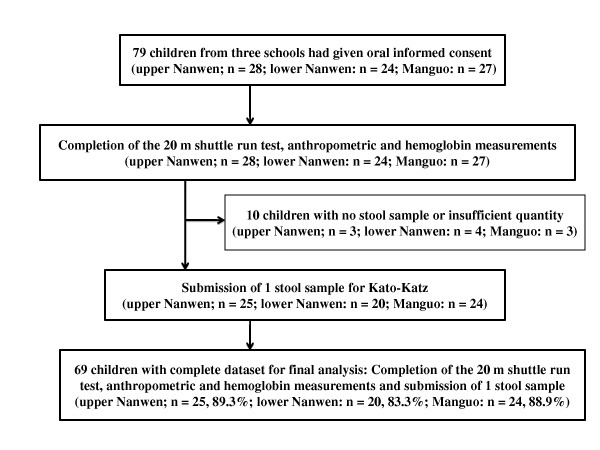
**Study cohort and compliance of school-aged children from three Bulang villages, Yunnan province, P.R. China in mid-2011**.

All 79 children potentially available for the study received oral informed consent from their parents/guardians to participate in the study. All of them completed the 20-m shuttle run test and anthropometric and haemoglobin measurements. Sufficiently large stool samples for diagnostic work-up were provided by 69 children, resulting in an overall compliance of 87% (Figure 1). Subsequent analyses are based on this cohort of 69 children who had complete data records.

There were 40 girls (58%) and the median age of the study cohort was 11 years (range: 8-15 years). No significant difference was observed between the three villages with regard to the age distribution and sex ratio (both *P *> 0.05). All children were of Bulang ethnicity, and came from families with farming as their main source of income.

### STH infection status

Fifty-nine out of the 69 children (86%) were infected with at least one intestinal helminth species. Infection prevalences of *T. trichiura, A. lumbricoides *and hookworm were 81%, 44% and 6%, respectively. In addition, one infection with *Taenia *spp. was identified. Stratification by sex revealed that boys had higher mean EPG values than girls for all helminth species even though in terms of prevalence, fewer males were infected (Table 1). In addition, the odds of being infected with STHs decreased by 37% when age increased by a year. Furthermore, the prevalence of *T. trichiura *infection was significantly higher among younger children (8-11 years) compared to their older counterparts (12-15 years) (*P *= 0.010) (Table 1). Multiparasitism was common: slightly more than half of the infected children (51%) harboured dual-species infections (mostly *A. lumbricoides *plus *T. trichiura*). One triple-species infection was detected; the remaining 48% of the children were infected by only one species.

**Table 1:** Prevalence and intensity of soil-transmitted helminth infection (mean of duplicate Kato-Katz thick smears) in 69 school-aged Bulang children from Yunnan province, P.R. China in mid-2011, stratified by sex and age group.

		Sex			Age (years)			
			
		Male (N^a ^= 29)	Female (N^a ^= 40)		8-10 (N^a ^= 22)	11-12 (N^a ^= 33)	13-15 (N^a ^= 14)	
			
		n^b ^(%)	n^b ^(%)	*P - *value^c^	n^b ^(%)	n^b ^(%)	n^b ^(%)	*P - *value
***T. trichiura***	**Overall prevalence: **81%							
	**Prevalence**	23 (79)	33 (83)	0.738	19 (86)	29 (88)	8 (57)	0.036
	**Infection intensity**^d^							
	Mean EPG; SE^e^	419; 582	350; 506	0.565	486; 643	374; 515	140; 162	0.194
	Light (1-999)	20 (69)	29 (73)		16 (73)	25 (76)	8 (57)	
	Moderate (1000-9999)	3 (10)	4 (10)		3 (14)	4 (12)	n.r.	

***A. lumbricoides***	**Overall prevalence: **44%							
	**Prevalence**	9 (31)	21 (53)	0.076	13 (59)	12 (36)	5 (36)	0.201
	**Infection intensity**^d^							
	Mean EPG; SE^e^	30,719; 60,310	12,783; 29,677	0.701	23,557; 42,666	16,694; 48,090	7670; 11,1856	0.331
	Light (1-4999)	6 (21)	13 (33)		7 (32)	9 (27)	3 (21)	
	Moderate (5000-49,999)	1 (3.5)	7 (18)		4 (18)	2 (6.1)	2 (14)	
	Heavy (≥ 50,000)	2 (6.9)	1 (2.5)		2 (9.1)	1 (3.0)	n.r.	

**Hookworm**	**Overall prevalence: **6%							
	**Prevalence**	3 (10)	1 (2.5)	0.302	n.r.	3 (9.1)	1 (7.1)	0.409
	**Infection intensity**^d^							
	Mean EPG; SE^e^	56; 66	12; n.a.	0.346	n.r.	56; 66	12; n.a.	0.371
	Light (1-1999)	3 (10)	1 (2.5)			3 (9.1)	1 (7.1)	

***Taenia *spp**.	**Prevalence**	n.r.	1 (2.5)	1.000	n.r.	1 (3.0)	n.r.	1.000

^a^N: total sample size

^b^n: number of infected individuals

^c^*P*-values are calculated using χ^2^, Fisher’s exact, Wilcoxon rank-sum or Kruskal-Wallis test, as appropriate

^d^Stratified according to WHO guidelines

^e^Arithmetic mean among the infected; standard error (SE)

n.a.: not applicable; n.r.: not represented

### Anthropometric indices and haemoglobin in relation to parasitological status

The mean height, weight and BMI of the study cohort were 134.7 cm, 31.2 kg and 16.9 kg m^-2^, respectively. As shown in Table 2, children infected with *T. trichiura *had significantly lower body weight, height and BMI than their peers who were *T. trichiura*-free. Stunting was observed in 59% of the participants, and 5.8% of them were wasted. No statistically significant associations between these two indicators and STH infection status were observed. The mean haemoglobin level was 15.7 g dl^-1 ^with no anaemia observed regardless of children's STH infection status.

**Table 2:** Anthropometric indicators and haemoglobin concentration, in relation to parasitological status, of 69 school-aged Bulang children from Yunnan province, P.R. China in mid-2011.

	*T. trichiura*			*A. lumbricoides*			Hookworm		
		
	Non-infected (n = 13)	Infected (n = 56)	*P* - value^a^	Non-infected (n = 39)	Infected (n = 30)	*P *- value^a^	Non-infected (n = 65)	Infected (n = 4)	*P *- value^a^
**Anthropometric**									
Mean weight [kg] (range)	36.0 (21.3-47.9)	30.1 (16.8-53.7)	0.020	31.9 (18.1-53.7)	30.3 (16.8-47.9)	0.433	30.9 (16.8-53.7)	37.2 (29.4-42.9)	0.138
Mean height [cm] (range)	140.5 (118.0-154.9)	133.3 (110.3-155.9)	0.037	136.6 (116.5-155.9)	132.2 (110.3-151.1)	0.111	134.2 (110.3-155.9)	142.8 (134.0-151.5)	0.147
Mean BMI [kg m^-2^] (range)	18.0 (15.3-21.8)	16.6 (13.3-23.5)	0.034	16.8 (13.3-23.5)	17.0 (13.8-21.5)	0.729	16.8 (13.3-23.5)	18.2 (16.3-21.5)	0.212
% wasted^b^	n.r.	7.1	1.000	10.3	n.r.	0.127	6.5	n.r.	1.000
% stunted^c^	53.9	60.7	0.650	56.4	63.3	0.562	60.0	50.0	0.693

**Haemotologic**									
Mean haemoglobin [g dl^-1^] (range)	14.1 (11.6-17.5)	16.1 (12.5-20.1)	< 0.001	15.3 (11.6-18.9)	16.2 (12.5-20.1)	0.043	13.4 (11.0-15.6)	14.3 (13.4-15.2)	0.147

^a^* P*-values are calculated using two-sample t test, Wilcoxon rank-sum, χ^2^ or Fisher’s exact test, as appropriate.

^b^ Wasting is defined as ≤ -2 BAZ score.

^c^ Stunting is defined as ≤ -2 HAZ score

n.r.: not represented

### Physical fitness in relation to parasitological status

The mean VO_2 _max estimates of boys infected with *A. lumbricoides, T. trichiura *and hookworm were 44.5, 45.1 and 44.4 ml kg^-1 ^min^-1^, respectively. A considerably higher mean VO_2 _max estimate was determined for helminth-free boys (48.2 ml kg^-1 ^min^-1^). For girls, no such differences were observed. However, the two girls infected with hookworm or *Taenia *spp. had lower VO_2 _max estimates (37.8 and 40.3 ml kg^-1 ^min^-1^, respectively) than helminth-free girls (41.8 ml kg^-1 ^min^-1^). After stratification by age group, children infected with *A. lumbricoides *and *T. trichiura *had reduced mean VO_2 _max estimates compared to their peers in the same age class who were not infected with either parasite. A statistically significant difference (*P *= 0.027) was observed between 11- to 12-year-old children infected with *A. lumbricoides *(42.0 ml kg^-1 ^min^-1^) and their counterparts not infected with this parasite (44.2 ml kg^-1 ^min^-1^; Table 3).

**Table 3:** Mean VO_2 _max estimates^a ^(ml kg^-1 ^min^-1^), in relation to parasitological status, of 69 Bulang primary school children from Yunnan province, P.R. China in mid-2011, stratified by sex and age group.

	*T. trichiura*			*A. lumbricoides*			Hookworm		
		
	Non-infected (n = 13)	Infected (n = 56)	*P - *value^b^	Non-infected (n = 39)	Infected (n = 30)	*P - *value^b^	Non-infected (n = 65)	Infected (n = 4)	*P *- value^b^
**Sex**									
**Male (n = 29)**	47.7 (44.1-51.3)	45.1 (43.7-46.5)	0.097	46.1 (44.5-47.8)	44.5 (42.2-46.9)	0.242	45.8 (44.4-47.2)	44.4 (34.9-53.9)	0.504
**Female (n = 40)**	41.9 (40.5-44.3)	43.3 (42.2-44.3)	0.263	42.7 (41.3-44.2)	43.3 (42.1-44.6)	0.513	43.2 (42.3-44.1)	37.8 (n.a.)	n.a

**Age (years)**									
**8-10 (n = 22)**	48.7 (36.8-60.5)	46.5 (45.5-47.5)	0.171	47.8 (45.3-50.3)	46.1 (45.1-47.2)	0.136	46.8 (45.7-47.9)	n.r.	n.a.
**11-12 (n = 33)**	43.5 (41.5-45.5)	43.4 (42.3-44.5)	0.935	44.2 (42.9-45.4)	42.0 (40.9-43.2)	0.027	43.5 (42.6-44.4)	42.0 (28.9-55.1)	0.358
**13-15 (n = 14)**	43.3 (39.5-47.1)	40.4 (38.5-42.4)	0.100	41.9 (39.1-44.8)	41.2 (38.5-43.9)	0.701	41.4 (39.5-43.3)	44.0 (n.a.)	n.a.

^a ^All mean VO_2 _estimates are expressed in ml kg^-1 ^min^-1^, with 95% confidence intervals in brackets when appropriate

^b ^All *P*-values calculated using a two-sample *t*-test

n.a.: not applicable; n.r.: not represented

In terms of number of 20-m laps successfully completed by the participants, both boys and girls infected with *A. lumbricoides *and *T. trichiura *achieved less laps than their non-infected counterparts (Table 4). This difference reached statistical significance in boys with or without *A. lumbricoides *infection (27.4 laps *versus *36.1 laps, *P *= 0.045). Similarly, boys infected with *T. trichiura *completed 30.5 laps while their counterparts not infected with this parasite completed significantly more laps (44.5 laps, *P *= 0.003). Stratified by age group, significant differences were observed among children aged 11-12 years depending on their infection status with *A. lumbricoides *(21.3 laps among infected and 28.7 laps among non-infected children, *P *= 0.019), and among children aged 8-10 years and their *T. trichiura *infection status (23.9 laps among infected and 35.3 laps among non-infected, *P *= 0.028).

**Table 4:** Mean number of 20-m laps^a ^completed by 69 school-aged Bulang children from Yunnan province, P.R. China in mid-2011, in relation to parasitological status, stratified by sex and age group.

	*T. trichiura*			*A. lumbricoides*			Hookworm		
		
	Non-infected (n = 13)	Infected (n = 56)	***P* - value^b^**	Non-infected (n = 39)	Infected (n = 30)	***P* - value^b^**	Non-infected (n = 65)	Infected (n = 4)	***P *- value^b^**
**Sex**									
**Male (n = 29)**	44.5 (35.8-53.2)	30.5 (26.4-34.6)	0.003	36.1 (31.1-41.0)	27.4 (20.4-34.5)	0.045	33.0 (28.7-37.3)	36.7 (29.3-37.5)	0.587
**Female (n = 40)**	26.0 (20.7-31.3)	22.3 (20.7-23.9)	0.063	24.4 (21.9-26.8)	21.7 (19.7-23.6)	0.076	23.3 (21.8-24.7)	12.0 (n.a.)	n.a

**Age (years)**									
**8-10 (n = 22)**	35.3 (0-73.8)	23.9 (21.7-29.3)	0.028	29.6 (21.6-37.6)	22.7 (19.2-26.2)	0.061	25.5 (21.7-29.3)	n.r.	n.a.
**11-12 (n = 33)**	28.3 (17.6-38.9)	25.7 (22.2-29.2)	0.595	28.7 (24.3-33.0)	21.3 (18.3-24.4)	0.019	25.9 (23.0-28.8)	27.0 (0-73.5)	0.841
**13-15 (n = 14)**	38.3 (25.2-51.5)	29.6 (24.2-35.1)	0.115	35.1 (26.7-43.5)	30.2 (19.3-41.1)	0.408	32.8 (26.5-39.0)	41.0 (n.a.)	n.a.

^a ^Mean number of laps completed with 95% confidence intervals in brackets when appropriate

^b ^All *P*-values calculated using a two-sample *t*-test

n.a., not applicable; n.r., not represented All *P*-values calculated using a two-sample *t*-test

According to the multiple linear regression models presented in Tables 5 and 6, sex, stunting and *T. trichiura *infection showed significant negative correlations with mean VO_2 _max estimates and number of 20-m laps completed. *T. trichiura *infection in children lowered the mean VO_2 _max estimate by 1.94 ml kg^-1 ^min^-1 ^(*P *= 0.005) and resulted in 6.1 fewer laps compared to children without *T. trichiura *infection (*P *= 0.004). In addition, the mean VO_2 _max estimate of stunted children was lowered by 1.63 ml kg^-1 ^min^-1 ^(*P *= 0.002) and they completed 5.3 laps less compared to children of standard stature (*P*-value = 0.001).

**Table 5:** Multiple linear regression model with mean VO_2 _max estimates (ml kg^-1 ^min^-1^) as outcome and age, sex, stunting and infection status as explanatory variables. Data is derived from 69 Bulang primary school children from Yunnan province, P.R. China, in mid-2011.

(I)	**Multiple linear regression**^a^		
	
Explanatory variables	Coefficient	95% confidence interval	*P* - value
Age (in years)	-1.37	-1.67 to -1.08	< 0.001
Sex (reference: male)	-2.36	-3.38 to -1.35	< 0.001
Stunting (reference: not stunted)	-1.63	-2.63 to -0.63	0.002
*A. lumbricoides *(reference: not infected)	-0.98	-2.03 to 0.07	0.066
*T. trichiura *(reference: not infected)	-1.94	-3.26 to -0.62	0.005

^a ^Only explanatory variables that have shown a significant difference in the descriptive statistics were included.

Key indicators of model: F (5, 63) = 23.97; *p *< 0.001; R-squared = 0.66

**Table 6:** Multiple linear regression models with number of 20-m laps completed as outcome and age, sex, stunting and infection status as explanatory variables. Data is derived from 69 Bulang primary school children from Yunnan province, P.R. China, in mid-2011.

(II)	Multiple linear regression^a^		
	
Explanatory variables	Coefficient	95% confidence interval	*P* - value
Age (in years)	0.86	-0.07 to 1.79	0.069
Sex (reference: male)	-9.89	-13.08 to -6.70	< 0.001
Stunting (reference: not stunted)	-5.32	-8.48 to -2.17	0.001
*A. lumbricoides *(reference: not infected)	-2.83	-6.12 to 0.46	0.090
*T. trichiura *(reference: not infected)	-6.14	-10.29 to -1.99	0.004

^a ^Only explanatory variables that have showed significant difference in the descriptive statistics were included.

Key indicators of model: F (5, 63) = 16.94; *P *< 0.001; R-squared = 0.57

## Discussion

In this cohort of school-aged children belonging to the Bulang ethnic minority in Yunnan province, southwest P.R. China, we found significantly impaired physical fitness due to STH infections. Indeed, children who were infected with *T. trichiura *or who were stunted had significantly lower mean VO_2 _max estimates and completed significantly fewer 20-m laps in a shuttle run test than children without *T. trichiura *infection and who were of standard stature. An earlier study also conducted in a Bulang community located in close proximity to the current setting revealed prevalences for each of the three STHs above 85% with almost two-thirds (62.3%) of the participants harbouring three helminth species concurrently [23]. We found somewhat lower prevalences, most likely as a result of recent STH control efforts. Moreover, in the previous investigation a suite of diagnostic methods was employed, which increased the diagnostic sensitivity. The high prevalence of stunting, regardless of the present STH infection status, might indicate that virtually all children have experienced STH infections at some point in their life. This is supported by the high prevalences reported previously, and would mean that it is difficult to draw conclusions from comparisons of long-term growth indicators with current infection status, as it is done in a cross-sectional study design. Longitudinal monitoring would be required instead. In terms of infection intensities and multiparasitism, no clear relationship was observed between infection intensity and physical fitness, but an increase in physical fitness impairment was observed in children with dual or triple species infection as compared to children with single species infections. Due to the small overall sample size, very small groups resulted after stratification by species-specific infection intensity and multiparasitism. Therefore, results should not be over-interpreted.

Assessments of physical fitness in relation to STH infection status have mainly relied on the HST [17] and the 20-m shuttle run test [22]. During pilot-testing, we found that it was difficult to standardize the HST across the different villages and that children took a longer time to learn and perform this test properly as compared to the 20-m shuttle run test. Thus, the 20-m shuttle run test was chosen for the current study. In the 20-m shuttle run test, the particular pace at which the signals are sounded for a min is also termed as a stage and within a stage, there are several sub-stages to complete for that pace. When estimating the mean VO_2 _max, using the equation put forth by Léger *et al. *[22], these sub-stages are not taken into account. We argue that sub-stages might not be of significance in healthy children of normal growth but given that the burden due to STH infections in children is still ambiguous, it might be important to consider such subtleties when studying the difference in physical fitness between infected and non-infected children. Hence, the number of completed 20-m laps was used as an additional outcome measure on top of the mean VO_2 _max estimate. We speculate that this simple indicator could serve as a straightforward measure of physical fitness [31].

There are some limitations to this study. First, our study was designed as a cross-sectional survey and as such could only identify associations rather than causality. Second, the overall sample size was small. Third, only a single stool sample was collected from each participant. Hence, some STH infections, particularly those of light intensity, were probably missed, as seen in other studies where multiple stool samples and a combination of diagnostic methods had been employed [23,32,33].

Despite these limitations, as a proof-of-concept, our study has shown the feasibility of conducting physical fitness testing along with stool examination, anthropometric measurements and determining haemoglobin levels in an ethnic minority group of P.R. China. Hence, our study confirms previous experiences in different African settings, where school-aged children were also receptive to physical fitness tests to determine whether physical fitness was negatively impacted by helminth infections [20,21].

Most Bulang families are engaged in agriculture. It is therefore conceivable that reduced physical fitness translates into lowered work productivity or increased exhaustion. The high prevalence of STHs and the marked differences in physical fitness and anthropometric measures between infected and non-infected children, which in certain cases reached statistical significance even in our small sample, are of considerable concern. These preliminary findings warrant larger follow-up studies. We have launched a new study with a larger cohort of 9- to 12-year-old Bulang children. In a baseline survey, children are rigorously diagnosed for STH infections, followed by random allocation of infected children into a treatment group (triple dose albendazole) or a placebo group, and monitoring of physical fitness over a 7-month period post-treatment.

## Conclusion

In summary, our study provided a snapshot of the effect of STH infections on the growth and physical fitness of school-aged children in an ethnic minority group of rural southwest P.R. China. Our preliminary results suggest that children who were stunted or infected with *T. trichiura *had reduced physical fitness. The current study confirmed the feasibility of implementing physical fitness tests in a rural, resource-constraint setting, and provided the basis for a more elaborate study, currently ongoing, to investigate the effect of de-worming on physical fitness in school-aged children.

## Competing interests

The authors declare that they have no competing interests.

## Authors' contributions

PY designed and implemented the study, entered, analyzed and interpreted the data and prepared the manuscript; ZWD, RC, LPZ, FWW, JW, XZW, HZ assisted in the design and implementation of study; XNZ designed the study, supervised its implementation and revised the manuscript; JU designed the study, interpreted the data and revised the manuscript; PS designed the study, facilitated its implementation, supervised PY, interpreted the data and revised the manuscript. All authors read and approved the final manuscript prior to submission.
